# Catechol-*O*-Methyltransferase Gene Polymorphism Is Associated with Skeletal Muscle Properties in Older Women Alone and Together with Physical Activity

**DOI:** 10.1371/journal.pone.0001819

**Published:** 2008-03-19

**Authors:** Paula H. A. Ronkainen, Eija Pöllänen, Timo Törmäkangas, Kristina Tiainen, Markku Koskenvuo, Jaakko Kaprio, Taina Rantanen, Sarianna Sipilä, Vuokko Kovanen

**Affiliations:** 1 Department of Health Sciences, University of Jyväskylä, Jyväskylä, Finland; 2 The Finnish Centre for Interdisciplinary Gerontology, University of Jyväskylä, Jyväskylä, Finland; 3 Department of Public Health, University of Turku, Turku, Finland; 4 Department of Public Health, University of Helsinki, Helsinki, Finland; 5 Department of Mental Health and Alcohol Research, National Public Health Institute, Helsinki, Finland; Erasmus University Medical Center, Netherlands

## Abstract

**Background:**

Muscle strength declines on average by one percent annually from midlife on. In postmenopausal women this decrement coincides with a rapid decline in estrogen production. The genetics underlying the effects of estrogen on skeletal muscle remains unclear. In the present study, we examined whether polymorphisms within *COMT* and *ESR1* are associated with muscle properties and assessed their interaction and their combined effects with physical activity.

**Methodology/Principal Findings:**

A cross-sectional data analysis was conducted with 434 63-76-year-old women from the population-based Finnish Twin Study on Aging. Body anthropometry, muscle cross-sectional area (mCSA), isometric hand grip and knee extension strengths, and leg extension power were measured. COMT Val158Met and ESR1 PvuII genotypes were determined by the RFLP method. mCSA differed by COMT genotypes (p = 0.014) being significantly larger in LL than HL individuals in unadjusted (p = 0.001) and age- and height-adjusted model (p = 0.004). When physical activity and age were entered into GEE model, COMT genotype had a significant main effect (p = 0.038) on mCSA. Furthermore, sedentary individuals with the HH genotype had lower muscle mass, strength and power, but they also appeared to benefit the most from physical activity. No association of ESR1 PvuII polymorphism with any of the muscle outcomes was observed.

**Conclusions/Significance:**

The present study suggests that the COMT polymorphism, affecting the activity of the enzyme, is associated with muscle mass. Furthermore, sedentary individuals with potential high enzyme activity were the weakest group, but they may potentially benefit the most from physical activity. This observation elucidates the importance of both environmental and genetic factors in muscle properties.

## Introduction

One of the most important functions of skeletal muscle is voluntary movement. Muscle also serves as an amino acid reservoir and a site for various metabolic activities participating e.g. to the glucose metabolism of the whole body [1], [2]. Absolute muscle mass is again critical, when the body has to recover from critical illnesses or traumatic injuries [3]. From midlife on, muscle strength declines, approximately one percent annually [4]. This decline may eventually predispose people to mobility limitation, falls and bone fractures [5]–[7]. Individual differences in muscle phenotypes in old age may be explained by both environmental and genetic factors [8]–[12].

In women, menopause is characterized by rapid decline in the production of estrogen, an anabolic female sex hormone, and coincides with an accelerated deterioration in muscle performance [13], [14]. At phenotype level hormone replacement therapy (HRT) exerts positive effects on skeletal muscle in some of the randomized, controlled trials reported [15]–[18], whereas others have documented contradictory results [19]–[21]. Moreover, a combination of high-impact training and HRT usage has been reported to exceed the beneficial effects of HRT alone [15]. Furthermore, in animal studies estrogen has been shown to contribute to skeletal muscle growth via specific receptors [22], [23]. Given this putative role of estrogen in muscle function, genes related to estrogen action and metabolism are likely candidates contributing to the genetic component of muscle properties.

The synthesis and degradation of estrogens are mediated by several enzymes involved in multiple and complex metabolic pathways. After initial hydroxylation of estrogens by isoenzymes belonging to the cytochrome P450 family, they are further metabolized by catechol-*O*-methyltransferase (COMT) into more inactive methoxyestrogens. These *O*-methylated metabolites no longer bind to estrogen receptors (ESRs) [24], which mediate the effects of estrogen in target tissues [25]. A functional G to A polymorphism in the fourth exon of *COMT* gene results in a valine to methionine amino acid transition at codon 158 (COMT Val158Met polymorphism) leading to thermolability [26], [27] and lower activity of the enzyme. This could hence increase the availability of estrogen and induce the anabolic effects of this hormone on target tissues such as skeletal muscle. COMT represents an intracellular enzyme present in a variety of tissues [28]. Previous studies with men [29], [30], early pubertal girls [31], as well as premenopausal [32] or postmenopausal women [33]–[35] have reported contradictory results, whether this polymorphism is actually associated with estrogen levels or not. To our knowledge, no studies have been published, in which the relationship of this polymorphism with skeletal muscle characteristics has been investigated.

ESR1 was recently shown to be expressed in human skeletal muscle [36] implying that skeletal muscle is sensitive to estrogen signaling, albeit to date the overall effects of estrogen on skeletal muscle remain poorly understood. PvuII polymorphism in the first intron of *ESR1* gene (ESR1 PvuII polymorphism) identifies a nucleotide T to C transition resulting in the loss of PvuII restriction site [37], which has been suggested to affect the magnitude of ESR1 transcription or production of various ESR1 isoforms [38]. More precisely, the C variant, also denoted as the P allele, is suggested to lead to the amplification of ESR1 transcription. In previous reports, no association of this polymorphic site with isometric muscle strength has been found [39], [40].

Theoretically, polymorphisms residing in genes related to estradiol metabolism and action, in this case *COMT* and *ESR1*, may have shared effects on estradiol signaling in target tissues. More precisely, a polymorphism affecting the activity of COMT may directly or indirectly modulate the amount of estradiol available to be bound by membrane-bound or intracellular estrogen receptors, whereas a polymorphism potentially modulating the amount of ESR1 transcript may further affect the availability of these receptors. On the other hand, the two estrogen-related polymorphisms under investigation may act in conjunction with physical activity resulting in a specific muscle phenotype. Our aim was to determine whether the two estrogenic polymorphisms, COMT Val158Met and ESR1 PvuII, are associated with serum estradiol levels and skeletal muscle phenotypes or not. Furthermore, we studied the interaction of these polymorphisms and their interaction with physical activity.

## Methods

### Study subjects

This study is a part of the Finnish Twin Study on Aging (FITSA), which investigates the genetic and environmental effects on the disablement process in older female twins. The detailed study design, including selection procedures, determination of zygosity and description of the participants, has been reported elsewhere [10]. Briefly, participants were recruited from the Finnish Twin Cohort [41], [42] consisting of 13 888 twin pairs. 103 monozygotic and 114 dizygotic female twin pairs (total n = 434 subjects) aged 63–76 years (mean 68.6 years, SE 0.16) were invited to laboratory investigations performed in Jyväskylä during 2000–2001. Shared willingness to participate obtained from both sisters was a prerequisite for recruiting. Zygosity was determined with a battery of ten highly polymorphic gene markers using DNA extracted from a venous blood sample. The Ethics Committee of the Central Finland Hospital approved the study and it was conducted according to the guidelines in The Declaration of Helsinki. The subjects signed an informed consent before participating in the measurements.

In the whole study sample the prevalence of various diseases potentially effecting muscle properties were: 12 % for coronary heart disease, 8 % for asthma, 7 % for cerebrovascular disease, 6 % for type 2 diabetes, 4 % for rheumatoid arthritis, 29 % for knee, 14 % for hip and 12 % for foot and ankle osteoarthritis. 67 % of all the subjects had never used HRT, whereas 14 % were former users and 19 % current users. Moreover, 3 % of the subjects in the entire population were current cortisol users and 12 % former users, whereas 85 % had never been under long-term cortisol treatment. Subjects with the above-mentioned diseases and different HRT or cortisol status were equally distributed between genotypes.

### Phenotyping

#### Hormone measurements

Nonfasting blood samples were collected between 0830 and 0930 hours and sera stored at −70°C for later analysis. Total serum 17β-estradiol was determined by competitive immunoenzymatic colorimetric assay (NovaTec Immunodiagnostica GmbH, Dietzenbach, Germany) and serum sex hormone binding globulin (SHBG) levels using solid-phase, chemiluminescent immunometric assay (Immulite® 1000 SHBG, DPC [Diagnostic Products Corporation], Los Angeles, USA). Free estradiol levels were calculated from 17β-estradiol and SHBG levels according to a previously presented method [43], [44]. In our laboratory, intra- and interassay coefficient of variations (CVs) were 3.2 % and 3.2 % for 17β-estradiol and 2.3 % and 6.5 % for SHBG, respectively.

#### Body anthropometry

Body mass and body height were measured using standard procedures. Lean body mass and total body fat were assessed by bioelectrical impedance (Spectrum II; RJL Systems, Detroit, MI, USA). The CV in our laboratory has been less than 2 % for lean body mass and less than 3 % for body fat [45]. Lower leg muscle cross-sectional area (mCSA), which gives an estimate of muscle mass, from the dominant hand side was assessed by peripheral quantitative computed tomography (pQCT, XCT-2000, Stratec Medizintechnik, Pforzheim, Germany). Tomography slices of two millimeters were obtained at 55 % upwards from the joint surface of the distal tibia. The whole mCSA of lower leg, i.e. the tissue area excluding subcutaneous fat and bones, was determined by Bonalyse 1.3 (Commit; Ltd, Espoo, Finland). CV for mCSA in our laboratory was 1 %.

#### Muscle strength and power

Maximal isometric strength, defined as the maximum voluntary contraction performed at a specific joint ankle against unyielding resistance, was measured for knee extension and hand grip. Maximal isometric hand grip and knee extension strengths were measured on the dominant side in a sitting position using an adjustable dynamometer chair (Good Strength, Metitur, Jyväskylä, Finland). After familiarization with the test, three to five maximal efforts separated by a one-minute interval were conducted. Mid-life maximal isometric hand grip strength, strength of a non-bearing limb, has been reported to correlate with strength of other muscle groups thus being a good indicator of overall strength [46], and also to be highly predictive of mortality and functional disability in later life [47], [48]. Knee extension strength, on the other hand, represents the strength of a bearing limb, potentially prone to physical exercise or disuse. Knee extension strength is important for functional capacity as decline in strength together with poor balance is reported to predict severe walking disability [49]. Leg extensor power of single leg was assessed according to published guidelines [50] using the Leg Extensor Power Rig (Nottingham, UK). Leg extension power measures the ability of the neuromuscular system to produce the greatest possible force as fast as possible. After two to three practice trials, five to nine maximal efforts were conducted. In all measurements, the best performance with the highest value was accepted as the result for each subject. CVs between two consecutive measurements have in our laboratory been 6 % for knee extension and hand grip strengths [51] and was 8 % for leg extension power in the present study. Physician evaluated possible contraindications for muscle strength and power measurements. All the subjects were able to perform at least one of the above-mentioned measurements.

#### Physical activity

Information concerning physical activity was collected using the scale of Grimby [52] with slight modifications. The participants were categorized on the basis of their self-reported physical activity as sedentary group (no other activities, but at the most light walking ≤2 times/week), moderately active (walking or other light exercise at least 3 times/week, but no other more intensive activities) and active (moderate or vigorous exercise at least 3 times/week). Of the entire population, 32 % were sedentary (sed), 48 % moderately active (mod) and 20 % active (act) according to this division. Subjects with various physical activity levels were equally distributed within the genotypes.

### Genotyping

Genomic DNA was extracted from EDTA-anticoagulated whole blood according to standard procedures (PUREGENE® Kit, Gentra Systems Inc., Minneapolis, USA). Genotyping for Val158Met and PvuII polymorphisms was performed using PCR (thermal cycler: Eppendorf® Mastercycler® gradient, Eppendorf, Boulder, CO, USA) followed by restriction fragment length polymorphism (RFLP) analysis [37], [53].

#### COMT Val158Met

The G to A transition at the 158^th^ codon in the *COMT* gene was determined by copying a 109-bp fragment with a primer pair (Oligomer Oy, Helsinki, Finland) including forward (5′-CTCATC ACCATC GAGATC AA) and reverse primers (5′-CCAGGT CTGACA ACGGGT CA) [54]. PCR reaction mixture of 15 µl contained 35 ng of DNA, 0.13 µM of both primers, 0.4 mM of dNTP mix (Eppendorf, Boulder, CO, USA) and 1,5 U of HotMaster Taq polymerase (Eppendorf, Boulder, CO, USA). PCR conditions included a pre-incubation period of 2 min at 95°C after which the DNA was subjected to 40 cycles of 95°C for 1 min, 54°C for 1 min and 72°C for 1 min followed by final extension step of 5 min at 72°C. The resulting PCR product was digested by adding 5 U of NlaIII restriction endonuclease (New England Biolabs, Ipswich, MA, USA) and incubated overnight at 37°C. The resulting fragments were separated in a 4.5 % agarose gel and the genotypes determined. Genotypes were coded as HH, HL and LL, in which capital H denotes the presence of valine and thus high activity allele, whereas L refers to the presence of methionine and the low activity allele.

#### ESR1 PvuII

In ESR1 PvuII genotyping a 373-bp PCR fragment was produced using a primer pair (Oligomer Oy, Helsinki, Finland) consisting of forward (5′-GATATC CAGGGT TATGTG GCA) and reverse primers (5′-TTACCT CTTGCC GTCTGT TGC) in a 10 µl reaction mixture containing 20 ng of DNA, 0.1 µM of both primers, 0.4 mM of dNTP mix (Eppendorf, Boulder, CO, USA) and 0.35 U of HotMaster Taq polymerase (Eppendorf, Boulder, CO, USA). PCR reaction was carried out with the following steps: preheating at 95°C for 2 min followed by 36 cycles, in which denaturation was performed at 94°C for 45 s, annealing for 45 s with gradually declining temperature (36 cycles with declining temperature from 67°C to 60°C) and extension at 72°C for 45 s. The PCR product was digested by adding 5 U of PvuII restriction endonuclease (Invitrogen™, Carlsbad, CA, USA) and incubated for two hours at 37°C. The digested products were further separated by electrophoresis in a 3 % agarose gel. Genotypes were determined due to resulting fragments and coded as PP, Pp and pp. Uppercase letters indicate the absence and lowercase letters the presence of a restriction site.

RFLP identification was carried out by two independent investigators from whom data on phenotypes was concealed. Genotyping was successfully performed in 423 for COMT Val158Met and 421 for ESR1 PvuII site out of 434 subjects. Reasons for missing determinations include insufficient amount of DNA or contamination of the blood sample.

### Statistical analyses

Hardy-Weinberg equilibrium was tested using the likelihood ratio test. Allele frequencies were determined by gene counting. All statistical models were constructed in SAS, version 9.1 using the generalized estimating equations approach (GEE), which allows taking into account the correlation between sisters within a twin pair. All outcome variables were normally distributed except for estradiol concentrations, which were skewed towards low concentrations and were considered to follow the gamma-distribution. Two types of single genotype models were constructed, one including the unadjusted main effects of the genotypes, and the other adjusted for age and height. To assess genotype-genotype and genotype-physical activity interactions a reference category was selected for the categorical predictor variables of physical activity (sedentary level), COMT (the HH genotype) and ESR1 Pvull (the pp genotype). Planned contrasts were used in comparing mean levels of each outcome variable between the predictor variable levels and their interactions against the reference category. Test-wise type I error rate was set at 0.05 in all analyses and partial correlation coefficients from the GEE model contrasts [55] were computed as estimates of effect size.

We hypothesized that subjects with assumed lower amount of circulating estradiol (HH genotype) and potential lower levels of ESR1 transcript (pp genotype) would be less responsive to estradiol and thus have worse muscle properties in comparison with other combinations. In the models including physical activity, subjects with potential low amount of circulating estradiol (HH genotype) or suggested low amount of ESR1 transcript (pp genotype) combined with sedentary life-style, were assumed to be weaker and have smaller muscles than other combinations. In our approach, reference groups were chosen according to these initial hypothesis and the mean values of other groups compared to that of the reference groups. The reference groups for the interaction effect were formed based on the combination of the main effect reference categories. The main effects of the two components of interest are always presented in contrast to the reference group.

## Results

### COMT Val158Met genotype

In our study population 79 subjects (18.0 %) were homozygous for the high activity allele (HH), 208 (47.9 %) heterozygotes (HL) and 137 (31.6 %) homozygous for the low active allele (LL). The allele frequencies were 0.43 for the H and 0.57 for the L allele. The genotype distribution of the entire cohort was in Hardy-Weinberg equilibrium (χ^2^ = 0.004, p = 0.95) suggesting that the subjects represented a homogeneous genetic background. Subject characteristics according to Val158Met genotypes are presented in Table 1. Physical characteristics and estradiol levels were similar in all Val158Met genotypes. However, mCSA was significantly larger in LL than HL individuals both in the unadjusted model (p = 0.001, data not shown) and after adjusting with age and height (p = 0.004, Table 1). No statistically significant association between Val158Met genotype and hand grip strength, knee extension strength or leg extension power was found.

**Table 1:** Body composition, hormone levels and muscle properties categorized according to COMT Val158Met genotypes.

Variable	COMT genotypes			p for trend
	HH (n = 79)	HL (n = 208)	LL (n = 136–137)	
Weight (kg)	70.0 (1.7)	70.3 (1.0)	70.0 (1.2)	0.972
Height (cm)	157.5 (0.87)	158.2 (0.53)	159.7 (0.66)	0.085
Body fat (kg)	24.0 (1.1)	24.5 (0.8)	23.6 (0.87)	0.763
Lean body mass (kg)	46.1 (0.68)	45.6 (0.36)	46.5 (0.47)	0.194
BMI (kg/m^2^)	28.2 (0.7)	28.2 (0.4)	27.6 (0.5)	0.565
Estradiol (nmol/l)	0.29 (0.051)	0.35 (0.054)	0.39 (0.069)	0.462
Free estradiol (nmol/l)	0.0059 (0.0008)	0.0073 (0.0010)	0.0083 (0.0015)	0.242
	**(n = 74–79)**	**(n = 193–208)**	**(n = 131–136)**	
Muscle CSA (mm^2^)*	5950.9 (109.8)	5880.6 (73.7)	6199.8 (91.5)	0.014§
Hand grip strength (N)*	190.6 (6.0)	189.3 (3.8)	194.0 (5.7)	0.763
Knee extension strength (N)*	352.5 (17.2)	322.5 (14.7)	343.4 (16.7)	0.092
Leg extension power (W)*	101.0 (4.2)	99.1 (2.6)	100.9 (3.4)	0.858

Data are mean (SE).

* Adjusted for age and height

§ Contrasts: COMT^LL^ vs. COMT^HL^ (p = 0.004); COMT^LL^ vs. COMT^HH^ (p = 0.078); COMT^HL^ vs. COMT^HH^ (p = 0.569)

### ESR1 PvuII genotype

The most common genotype was Pp (n = 187, 43.1 %), whereas pp genotype was more frequent (n = 144, 33.2 %) than PP (n = 90, 20.7 %). The allele frequencies were 0.44 and 0.56 for the P and p alleles, respectively. The genotypes were slightly out of Hardy-Weinberg equilibrium (χ^2^ = 3.943, p = 0.047) suggesting that our study sample may not be representative of the target population. Physical characteristics, including hormone levels, were similar in all PvuII genotypes. Furthermore, PvuII polymorphism was not associated with any of the measured muscle variables (Table 2).

**Table 2:** Body composition, hormone levels and muscle properties categorized according to ESR1 PvuII genotypes.

Variable	ESR1 genotypes			p for trend
	PP (n = 90)	Pp (n = 187)	pp (n = 144)	
Weight (kg)	70.3 (1.4)	69.6 (1.1)	70.7 (1.3)	0.797
Height (cm)	159.2 (0.88)	158.3 (0.58)	158.5 (0.63)	0.669
Body fat (kg)	24.3 (1.1)	23.7 (0.7)	24.6 (1.0)	0.664
Lean body mass (kg)	46.3 (0.52)	45.7 (0.44)	46.2 (0.44)	0.573
BMI (kg/m^2^)	27.8 (0.6)	27.9 (0.4)	28.3 (0.5)	0.813
Estradiol (nmol/l)	0.36 (0.08)	0.31 (0.04)	0.40 (0.08)	0.584
Free estradiol (nmol/l)	0.0075 (0.0017)	0.0062 (0.0006)	0.0086 (0.0017)	0.375
	**(n = 81–90)**	**(n = 177–187)**	**(n = 136–144)**	
Muscle CSA (mm^2^)*	6124.6 (121.5)	5927.3 (81.8)	6002.5 (97.4)	0.393
Hand grip strength (N)*	192.0 (6.3)	190.3 (4.4)	192.3 (5.2)	0.950
Knee extension strength (N)*	347.7 (17.8)	337.2 (14.8)	323.2 (15.1)	0.416
Leg extension power (W)*	99.2 (3.5)	98.3 (2.5)	103.5 (3.7)	0.494

Data are mean (SE).

* Adjusted for age and height

### Interaction of COMT and ESR1 polymorphisms with respect to muscle properties

We further studied whether ESR1 modified the effects of COMT. The results of age-adjusted models are shown in Table 3. In the model including COMT and ESR1 genotypes COMTVal158Met polymorphic site had a main effect on mCSA. More precisely, individuals with the HH genotype had significantly smaller muscle mass than LL subjects (p = 0.038). Furthermore, a significant interaction was present in knee extension strength between HH and LL subjects (p = 0.031). Here, the mean difference between the comparison and reference groups was −61.44, suggesting that an addition of two P alleles to LLpp genotype (LLpp→LLPP) leads to a lesser increase in knee extension strength in comparison to the HH genotype (HHpp→HHPP). Other interaction effects between COMT Val158Met and ESR1PvuII polymorphisms were not significant.

**Table 3:** Mean differences (Mdf), standard errors (SE), p values and partial correlations (r_Y⋅e_) for genetic effects in age-adjusted models including COMT Val158Met and ESR1 PvuII polymorphisms for muscle CSA, hand grip strength, knee extension strength and leg extension power.

Effect (reference group)		Muscle CSA				Hand grip strength				Knee extension strength				Leg extension power			
		Mdf	SE	p value	r_Y⋅e_	Mdf	SE	p value	r_Y⋅e_	Mdf	SE	p value	r_Y⋅e_	Mdf	SE	p value	r_Y⋅e_
Val158Met main	HL	−64.46	136.44	0.637	−0.034	0.76	7.37	0.918	0.007	−18.89	11.16	0.090	−0.124	−1.39	4.34	0.749	−0.023
effect (HH)	LL	307.15	147.78	**0.038**	0.148	6.94	9.11	0.446	0.054	−6.80	12.11	0.574	−0.041	1.86	5.16	0.718	0.026
PvuII main effect	Pp	−60.34	125.72	0.631	−0.034	−7.92	7.83	0.312	−0.072	−15.32	11.74	0.192	−0.096	−4.48	4.79	0.349	−0.067
(pp)	PP	165.45	151.92	0.276	0.078	−4.81	9.17	0.600	−0.037	−2.78	11.01	0.801	−0.019	−2.97	5.37	0.581	−0.040
Val158Met*PvuII	HLPp	−256.27	318.00	0.420	−0.058	16.13	18.57	0.385	0.062	14.90	28.24	0.598	0.039	−0.59	10.86	0.957	−0.004
interaction effect	HLPP	−304.91	381.91	0.425	−0.057	4.09	19.41	0.833	0.015	−15.94	26.63	0.549	−0.044	−6.98	11.41	0.540	−0.044
(HHpp)	LLPp	−572.52	324.83	0.078	−0.126	−17.25	20.40	0.398	−0.060	−9.03	31.12	0.772	−0.021	0.70	12.39	0.955	0.004
	LLPP	−302.04	380.25	0.427	−0.057	−44.14	25.18	0.080	−0.124	−61.44	28.44	**0.031**	−0.158	−18.20	13.14	0.166	−0.099

### Interaction of COMT or ESR1 polymorphism and physical activity with respect to muscle properties

**Figure 1:**
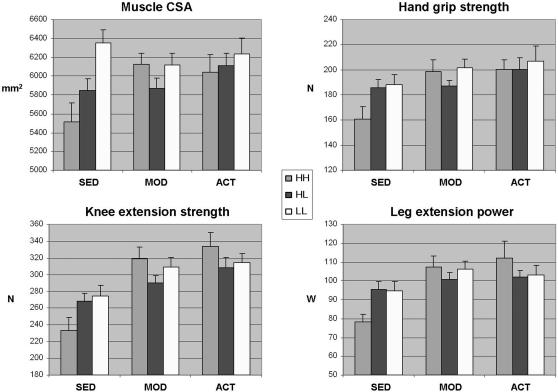
Muscle CSA, hand grip strength, knee extension strength and leg extension power according to COMT genotype and physical activity. Diagram presents the mean values (+SE) for CSA, hand grip strength, knee extension strength and leg extension power from GEE model according to COMT genotypes (HH, HL and LL) and physical activity (sed for sedentary, mod for moderately active and act for active). The model is adjusted with age. Results from statistical testing are shown in Table 4.

In further analyses we examined whether physical activity level modulates the effects of COMTVal158Met (Table 4 and Figure 1) or ESR1PvuII (Table 5) polymorphism on muscle properties. In the model including the COMT genotype, physical activity and age as explanatory variables, the genotype had a statistically significant main effect on mCSA; LL subjects were greater than HH subjects in their muscle size (p = 0.021). As expected, physical activity had a significant main effect on all the muscle strength and power variables (sedentary subjects were weaker than moderately active or active individuals, p≤0.004 for all comparisons), but the effect on mCSA was less clear (p≥0.078 for all comparisons). Significant interaction effects of the COMT genotype and physical activity were present in all muscle variables. In knee extension strength and leg extension power, all the interaction effects were statistically significant (p<0.05 for all comparisons). The mean differences imply that in all these comparisons, an increase in physical activity from sedentary to moderate or from sedentary to active level within the HH genotype, creates a larger increase in both knee extension strength and leg extension power than among HL or LL individuals (p≤0.045). This trend was also evident in mCSA, although the effect between HH and HL subjects was not statistically significant when sedentary and active individuals were compared (p = 0.41). In hand grip strength a significant interaction effect was observed only between HH and HL individuals, when sedentary subjects were compared to their moderate active counterparts (Mdf = −36.76, p = 0.011). In general, the mean values of sedentary HH subjects in all the measured muscle outcomes were lower than subjects with other genotype and/or physical activity level (Figure 1). Moderately active or active subjects with HH genotype, however, had comparable values to those of other genotypes. The partial correlations for the main and interaction effects indicate that the effect sizes were small (0.1) or moderate (0.3) in all significant effects.

**Table 4:** Mean differences (Mdf), standard errors (SE), p values and partial correlations (r_Y⋅e_) for genetic effects in age-adjusted models including COMT Val158Met polymorphism and physical activity for muscle CSA, hand grip strength, knee extension strength and leg extension power.

Effect (reference group)		Muscle CSA				Hand grip strength				Knee extension strength				Leg extension power			
		Mdf	SE	p value	r_Y⋅e_	Mdf	SE	p value	r_Y⋅e_	Mdf	SE	p value	r_Y⋅e_	Mdf	SE	p value	r_Y⋅e_
Val158Met main	HL	47.79	123.75	0.699	0.028	4.41	7.16	0.538	0.044	−4.87	10.62	0.646	−0.034	0.01	4.34	0.997	0.000
effect (HH)	LL	340.29	147.30	**0.021**	0.164	12.31	8.69	0.157	0.101	5.42	11.93	0.649	0.034	1.99	5.18	0.701	0.028
Physical activity	mod	135.35	105.35	0.199	0.092	17.58	6.05	**0.004**	0.204	48.19	9.97	<**0.001**	0.337	15.31	3.25	<**0.001**	0.323
main effect (sed)	act	223.79	127.19	0.078	0.126	24.22	7.12	**0.001**	0.237	60.91	10.67	<**0.001**	0.390	16.34	4.25	<**0.001**	0.268
Val158Met*physical	HLmod	−588.22	246.92	**0.017**	−0.169	−36.76	14.52	**0.011**	−0.178	−65.69	23.74	**0.006**	−0.201	−23.76	7.82	**0.002**	−0.215
activity interaction	HLact	−260.30	316.71	0.411	−0.059	−25.35	16.40	0.122	−0.110	−61.37	26.53	**0.021**	−0.169	−27.57	10.24	**0.007**	−0.191
effect (HHsed)	LLmod	−849.69	260.99	**0.001**	−0.228	−25.24	16.59	0.128	−0.108	−53.31	26.58	**0.045**	−0.147	−17.35	8.65	**0.045**	−0.144
	LLact	−644.78	331.07	**0.051**	−0.139	−21.59	17.69	0.222	−0.087	−62.35	28.02	**0.026**	−0.163	−25.42	11.26	**0.024**	−0.161

sed = sedentary

mod = moderately active

act = active

**Table 5:** Mean differences (Mdf), standard errors (SE), p values and partial correlations (r_Y⋅e_) for genetic effects in age-adjusted models including ESR1 PvuII polymorphism and physical activity for muscle CSA, hand grip strength, knee extension strength and leg extension power.

Effect (reference group)		Muscle CSA				Hand grip strength				Knee extension strength				Leg extension power			
		Mdf	SE	p value	r_Y⋅e_	Mdf	SE	p value	r_Y⋅e_	Mdf	SE	p value	r_Y⋅e_	Mdf	SE	p value	r_Y⋅e_
PvuII main effect	Pp	−13.08	127.74	0.918	−0.007	−4.90	7.41	0.508	0.047	−10.16	10.02	0.311	−0.075	−5.63	4.49	0.210	−0.090
(pp)	PP	170.25	162.36	0.294	0.075	−2.81	8.78	0.749	0.023	−2.82	10.45	0.787	−0.020	−3.23	5.23	0.537	−0.045
Physical activity	mod	27.06	116.03	0.816	0.017	17.05	5.91	**0.004**	0.202	37.90	9.40	<**0.001**	0.286	11.95	3.32	<**0.001**	0.252
main effect (sed)	act	115.03	132.59	0.386	0.062	26.27	6.87	<**0.001**	0.264	50.63	9.67	<**0.001**	0.362	12.53	3.80	**0.001**	0.232
PvuII*physical	Ppmod	−214.46	217.09	0.323	−0.071	−8.96	13.05	0.493	0.049	−13.22	19.57	0.499	−0.050	−9.99	7.44	0.179	−0.097
activity interaction	Ppact	133.93	234.63	0.568	0.041	−19.71	15.79	0.212	0.089	8.30	23.57	0.725	0.026	−7.58	9.25	0.412	−0.059
effect (ppsed)	PPmod	−270.12	316.90	0.394	−0.061	20.86	16.28	0.200	0.091	−22.52	23.62	0.340	−0.070	−8.07	8.90	0.364	−0.065
	PPact	−129.81	369.17	0.725	−0.025	12.89	18.20	0.479	0.051	−0.90	23.36	0.969	−0.003	1.32	10.05	0.896	0.010

sed = sedentary

mod = moderately active

act = activ

In the model including ESR1 genotype, physical activity and age as explanatory variables, physical activity had a main effect on muscle strength and power (sedentary subjects were weaker than moderately active or active individuals, p≤0.004, Table 5), but this effects was not observed in mCSA. Neither main effects of ESR1 nor interaction effects of ESR1 genotype and physical activity on any of the studied muscle properties were present.

## Discussion

In the present study we examined the contribution of inter-individual variation in two candidate genes involved in estrogen metabolism and action, *COMT* and *ESR1*, to skeletal muscle properties in older women. We hypothesized that variation in these genes, essentially Val158Met polymorphism within *COMT* and PvuII polymorphism within *ESR1*, alone or together with physical activity may, at least partly, modulate muscle mass and performance phenotypes in older women. Our results suggest that COMT Val158Met polymorphism is associated with muscle mass in that subjects with the LL genotype have significantly larger muscles than heterozygotes. Furthermore, within the subjects with HH genotype – leading to the presumed higher COMT activity – and sedentary life-style, lower levels of muscle mass, strength and power were observed than within other sedentary subjects or subjects with more active life-style.

Since the *O*-methylation of catechol estrogens by COMT occurs rapidly [24], Val158Met polymorphism within *COMT* may have significant contribution to circulating estrogen levels by mediating the activity of the enzyme. In the present study no statistically significant differences in total or free estradiol levels between different COMT genotypes were found, but there was a gradient, albeit not statistically significant, towards lower estradiol levels in HH individuals. Our subjects were postmenopausal women characterized by relatively low serum estradiol levels making their measurement with a direct assay difficult [56], thereby possibly affecting the accuracy of the results. From our data, the possibility that during the pre-menopausal years of our study subjects, i.e. the majority of their life-span, their estradiol levels may have been affected by COMT genotype, cannot be excluded. If so, their present muscle properties would also have been vulnerable to the effects of different hormone levels during these pre-menopausal years. Here, we can not rule out this period of their life, which certainly is evident in their present muscle phenotype. Thus far, contradictory results exist, whether this genotype can really affect free estradiol levels or not. In pubertal girls Val158Met genotype has been reported to affect free estradiol levels in that subjects with the low activity variant have higher levels of circulating estradiol [31]. Additionally, the same polymorphism has been demonstrated to result in higher bone mineral density in young men carrying the H allele compared to non-carriers [29] as well as increased non-vertebral fracture risk in elderly men with the L allele [30] without, however, an evident connection to free circulating estradiol levels. However, in postmenopausal women receiving an oral estradiol preparation, serum estradiol levels correlated significantly with Val158Met genotype [35], whereas no direct association between estradiol levels and Val158Met polymorphism was found elsewhere [32]–[34].

Our results suggest that Val158Met polymorphism affects muscle size such that LL genotype favors larger muscle cross-sectional area. This result is supported by a similar outcome in a study with early pubertal girls [31]. In previous studies muscle mass has been reported to be under rather strong genetic control [11], [12], whereas genetic effects explained muscle strength and power to a lesser extent [9], [10].

In our study sample, no association between PvuII polymorphism in *ESR1* and serum estradiol levels, or muscle structure or function was found. This supports the results from previous reports, in which no connection between this polymorphism and hand grip [39], [40] or quadriceps isometric strength [39] has been found. The biological significance of PvuII polymorphism is hitherto unclear. Loss of PvuII restriction site has been reported to result in a potential binding site for B-myb transcription factor and in some settings this further led to induced transcription of a reporter gene [38]. In addition to this possibility to influence the mRNA levels of *ESR1*, the connections of PvuII polymorphism to e.g. bone phenotypes recognized so far may be due to some unknown polymorphism residing in close proximity of and being in linkage disequilibrium with PvuII locus within *ESR1*. This polymorphic site within *ESR1*, or less likely within an adjacent gene, may affect bone, but not muscle properties supporting our results together with the findings from previous studies [39], [40].

In theory, polymorphisms within *COMT* and *ESR1* genes may act in concert by regulating the availability of estrogen and estrogen receptors, respectively. To the best of our knowledge, the present study is the first to examine, whether the genetic variation in ESR1 modulates the effects of COMT Val158Met on muscle properties. According to our results, COMT genotype has a main effect on muscle CSA regardless of the ESR1 genotype. Moreover, we observed an interaction effect of these polymorphisms on knee extension strength. This effect, however, seemed sporadic, given the small effect size and since other interaction effects were not present. Further studies are warranted in order to confirm this finding.

In the GEE model dissecting the interaction effects of COMT Val158Met polymorphism and physical activity on muscle properties a more clear gradient of the effects was observed. The HH subjects showed more variation in relation to physically active life-style compared to other genotypes as measured by knee extension strength and leg extension power. For example, the adjusted mean values in moderately active subjects with the HH genotype were 36.7 % higher in knee extension strength than in sedentary subjects with the same genotype, whereas within the HL genotype this difference was only 7.9 % in the favor of the moderately active subjects (Figure 1). In general, the sedentary subjects with the HH genotype were the weakest group, but those with the same genotype and more active life-style had comparable muscle properties to that of other genotypes with whichever level of activity. These data suggest that individuals with presumed low levels of circulating estradiol and thereby its minor effect on skeletal muscle can be prone to low muscle mass, strength and power, which may, however, be compensated for by physically active life-style. A clinical trial of muscle training among sedentary subjects with differing genotypes would be needed to confirm these observational data.

Physical activity had a significant main effect on muscle strength and power measures, in the models investigating the interaction effects between COMT or ESR1 genotype and physically active life-style. Here, both moderately active and active individuals were stronger than sedentary subjects. In muscle mass, however, the effect was less clear or absent. This observation shows that our assessment of physical activity level with the modified scale of Grimby was in accordance with our expectations in muscle performance variables, but not in muscle mass. This notion seems reasonable taken into account the general mode of physical activity in older subjects; physical activity in the ages around 60 and 70 in general is not hypertrophying that would be evident as an increased muscle mass, but rather includes various types of aerobic everyday activities affecting the properties of muscle performance. Our assessment was clearly able to differentiate sedentary individuals from more active ones in the model investigating the interaction of physical activity with the COMT genotype.

A limitation of the present study is the relatively small sample size, which may have also been selected towards rather healthy women creating a possible healthy population bias. Moreover, we present results from various measurements describing muscle strength. Our test battery includes variables presenting both isometric (hand grip and knee extension strength) and dynamic (leg extension power) muscle performance as well as measures from both lower and upper limbs. Furthermore, during isometric testing, the speed of muscle contraction is not as essential as in muscle power measurements. On the other hand, these data provide a multifaceted estimate of the effects of the chosen genotypes on whole body musculature. The results provided by our cross-sectional data set should be further confirmed in a follow-up study and, if possible, with a larger sample and an intervention trial to see, if the response to training is actually genotype-dependent.

In conclusion, the identification of the genetic susceptibility factors predisposing the elderly to impaired muscle performance could provide novel insights into the etiology of sarcopenia and would enable the recognition of those people at high risk of disability. Analysis of genetic variants, such as SNPs, represents a powerful approach to examine the role of candidate genes in the progression of this obviously multifactorial state. In the present study, we found an association between a polymorphism in the *COMT* gene and muscle mass. Furthermore, the interaction effect of this polymorphism and physical activity on muscle mass, strength and power elucidates the interplay of environmental and genetic factors in muscle properties and represents an example of how an unfavorable genetic background may perhaps be compensated for by healthy living habits, in this case physical activity. Overall, our results imply that *COMT* gene, related to the metabolism of estrogens, may be connected with muscle properties, albeit the exact mechanisms remain unknown.
